# Epicardial Fat in Heart Failure with Preserved Ejection Fraction Compared with Reduced Ejection Fraction

**DOI:** 10.3390/jcm13185533

**Published:** 2024-09-18

**Authors:** Gurwinder S. Sidhu, Simon W. Rabkin

**Affiliations:** 1 Faculty of Medicine, University of British Columbia, Vancouver, BC V6T 1Z3, Canada; gs1999@student.ubc.ca; 2 Department of Medicine, Division of Cardiology, University of British Columbia, Vancouver, BC V5Z 1M9, Canada

**Keywords:** HFpEF, HFrEF, epicardial fat, ventricular size, atrial size

## Abstract

**Background:** The role of epicardial adipose tissue (EAT) in heart failure with preserved ejection fraction (HFpEF) remains to be defined. **Methods:** A consecutive series of outpatients with chronic heart failure—heart failure with reduced ejection fraction (HFrEF) and HFpEF and/or diastolic dysfunction—had EAT assessed by echocardiographic measurement and related to indices of cardiac structure and function. **Results:** Epicardial fat thickness was significantly (*p* < 0.05) greater in HFpEF (*N* = 141) with a mean of 6.7 ± 1.6 mm compared with a mean of 5.1 ± 1.0 mm in HFrEF (*n* = 40). After adjusting for the relationship with BMI, in HFpEF, epicardial fat was significantly (*p* < 0.05) negatively correlated with left ventricular internal diameter end diastole (LVIDd), left ventricular internal diameter end systole (LVIDs), left ventricular (LV) end-diastolic volume (EDV) index, lateral e’, septal e’, right atrial (RA) volume index, and hemoglobin (Hgb). The association with Hgb was no longer significant after adjusting for the effect of age. HFpEF was associated with smaller LVIDd, LVIDs, LV EDV indexes, and left atrial (LA) and RA volume indexes. **Conclusions:** Epicardial fat is significantly (*p* < 0.05) greater in HFpEF than HFrEF. Epicardial fat is associated with smaller cardiac chamber sizes in HFpEF suggesting that epicardial fat acts as a constraint to cardiac dilation.

## 1. Introduction

Heart failure with preserved ejection fraction (HFpEF) is an important clinical entity as it represents almost fifty percent of all cases of heart failure and its prevalence is projected to increase as the population ages [1]. Despite recent evidence of the successful treatment of this condition with SGLT-2 inhibitors [2,3], there remains a significant morbidity and mortality with HFpEF. While diastolic dysfunction is the hallmark of most, if not all, patients with HFpEF [4], various mechanisms have been implicated in its pathogenesis ranging from abnormalities of inflammation, nitric oxide, endothelial dysfunction, extracellular matrix, HIF-1 alpha, and others [5,6,7,8]. 

A frontier of cardiac research examines epicardial fat and seeks to explain its role in inflammation, adiposity, and obesity in the pathogenesis of heart disease. Epicardial fat has similarities to other fat deposits in the body but also differs from other types of adipose tissue as reflected by significant differences in gene expression and in the production of an array of cytokines, adipokines, and inflammatory mediators [9,10,11,12,13]. With its close anatomic proximity to the heart and coronary arteries, epicardial fat has been proposed to play various roles in the regulation of cardiac homeostasis and in the pathogenesis of cardiac disease including diastolic dysfunction and HFpEF [14,15,16,17,18,19,20]. However, there have been variable or conflicting data on this relationship [21], with potential divergent roles for epicardial fat in HFpEF and heart failure with reduced ejection fraction (HFrEF), even suggesting that epicardial fat may be pathological in HFpEF and protective in HFrEF [17,19]. Considering the inconsistencies in results from studies of epicardial fat in HFpEF and the relative lack of comparative data with epicardial fat in HFrEF, we sought to explore the relationship between epicardial fat and cardiac function in HFpEF compared with HFrEF.

## 2. Methods

### 2.1. Study Type and Population

A consecutive series of patients attending an outpatient Cardiology Clinic was identified with echocardiographic evidence of HFrEF or HFpEF/diastolic dysfunction. The inclusion criteria for HFrEF patients was (i) a left ventricular ejection fraction (LVEF) less than or equal to 40% or (ii) a LVEF between 41% and 45% with a diagnosis of HFrEF previously documented on their chart. The inclusion criteria for HFpEF patients was (i) a LVEF greater than or equal to 50% or (ii) a LVEF between 45% and 49% with HFpEF documented on their chart and (iii) left ventricular diastolic dysfunction or (iv) elevated left ventricular filling pressures as defined using echocardiographic parameters from the American Society of Echocardiography guidelines [22]. The exclusion criteria for HFrEF and HFpEF patients were (i) severe valvular heart disease as defined using American Society of Echocardiography guidelines, (ii) heart valve replacement, (iii) or pericardial effusion of hemodynamic significance or tamponade [23,24]. BNP and NT-proBNP levels were not assessed for patient inclusion or exclusion given the variability in test ordering in this outpatient cohort and given that heart failure can be diagnosed in the absence of these investigations. This research study was granted ethics approval by our clinical research ethics board and all patients within this clinic had previously provided consent to research participation. The study population consisted of 181 patients ranging from ages of 40 to 98, of which, 40 patients were in the HFrEF group and 141 patients were in the HFpEF group.

### 2.2. Clinical Data

Data for this investigation were extracted from index echocardiogram reports and clinical documentation which were accessed using a local, clinic-based electronic medical record (EMR) system. The echocardiogram selected for evaluation was the most recent echocardiogram that satisfied the inclusion and exclusion criteria. Relevant demographic and clinical variables were extracted by a trained research assistant which included (i) age, gender, weight, and body surface area (BSA) reported on the patient’s index echocardiogram, (ii) systolic blood pressure, diastolic blood pressure, and heart rate reported on the patient’s index echocardiogram, (iii) relevant co-morbidities, (iv) cardiovascular medications, (v) hemoglobin, creatinine, and eGFR values, (vi) brain natriuretic peptide (BNP) and BNP prohormone, (vii) and echocardiographic measurements. Co-morbidities, cardiovascular medications, and lab values were included if they were documented on any of the patient’s medical records within 6 months of the index echocardiogram date. The only exception to this timeframe was BNP and BNP prohormone lab values which were considered within 12 months of the index echocardiogram date. For lab values with multiple repeat measurements over the specified timeframe, the measurement closest to the index echocardiogram date was used. 

### 2.3. Echocardiography

**Figure 1:**
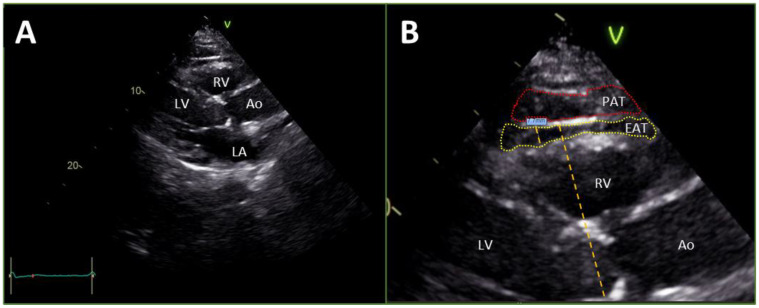
(**A**) The parasternal long axis view from a transthoracic echocardiogram of the heart at end-systole. Depth is quantified on the right axis in centimeters. (**B**) [An enlargement of image in A]The measurement of EAT thickness taken perpendicular to the free wall of the RV using the aortic annulus as an anatomical landmark for alignment and orientation (as shown with the orange dashed line). EAT is circumscribed with a yellow dashed line and PAT is circumscribed with a red dashed line. Ao, Aorta; EAT, epicardial adipose tissue; LA, left atrium; LV, left ventricle; PAT, pericardial adipose tissue; RV, right ventricle.

Epicardial fat thickness measurements were made from the 2D parasternal long-axis view of the transthoracic echocardiograms, perpendicular to the free wall of the right ventricle using the aortic annulus as an anatomic landmark for orientation and alignment as shown in Figure 1A,B [25,26]. To appreciate a consistent anatomical location for the purpose of measurement, epicardial fat was defined as a layer of echo-free tissue bordered by the myocardium below and visceral pericardium above [26]. Using the integrated ruler tool, the maximum epicardial fat thickness was measured at end-systole during the T wave. This approach for evaluating epicardial fat thickness has been validated against other methods of assessment of epicardial fat mass [27]. Three separate measurements were taken from three distinct cardiac cycles for each study patient and the mean value for epicardial fat thickness was computed and utilized for subsequent data analysis [25,26]. Echocardiographic measurements were made following standard procedures.

### 2.4. Data Analysis

Quantitative variables were computed as means with standard deviations while categorical variables were computed as frequencies with 95% confidence intervals. For quantitative variables with normal distributions based on the Shapiro–Wilk test, parametric hypothesis testing was performed using two-sample *t*-tests. In all other quantitative variables, non-parametric hypothesis testing was performed using Mann–Whitney tests. Chi-square tests were utilized to assess categorical variables. Pearson correlation coefficients were computed to identify linear correlations and the Spearman’s rank correlation coefficient were computed to identify non-linear correlations. Statistical analysis was performed using GraphPad Prism (Version 9.4.0, GraphPad Software, San Diego, CA, USA). Hypothesis testing used a pre-defined significance level of 0.05.

## 3. Results

The main clinical characteristics of the study population, as well as the comparison of these clinical characteristics between HFrEF and HFpEF patients, are presented in Table 1. The mean age was 72.0 ± 11.5 years with 59.7% of patients being male. There were no significant differences in age, sex, body weight, and body mass index (BMI) but HFrEF patients were taller and had a larger body surface area (BSA) than the HFpEF patients. A significantly greater prevalence of chronic kidney disease, coronary artery disease, and previous myocardial infarction was observed in HFrEF patients compared with HFpEF patients. There were no significant differences in the prevalence of hypertension, cigarette smoking, diabetes mellitus, dyslipidemia, or atrial fibrillation between the two groups. The use of angiotensin-converting enzyme inhibitors (ACEi)/angiotensin II receptor blockers (ARB), beta blockers, loop diuretics, vasodilators, cholesterol absorption inhibitors, sodium-glucose cotransporter-2 (SGLT2) inhibitors, and mineralocorticoid receptor antagonists (MRA) were more frequent in HFrEF patients than in HFpEF patients. The use of calcium channel blockers (CCB), thiazide diuretics, statins, and oral hypoglycemic agents did not differ significantly between the two groups. On average, HFrEF patients had significantly higher laboratory values for creatinine and BNP. HFpEF patients had significantly higher systolic blood pressure measurements while HFrEF patients had significantly higher heart rate readings.

**Figure 2:**
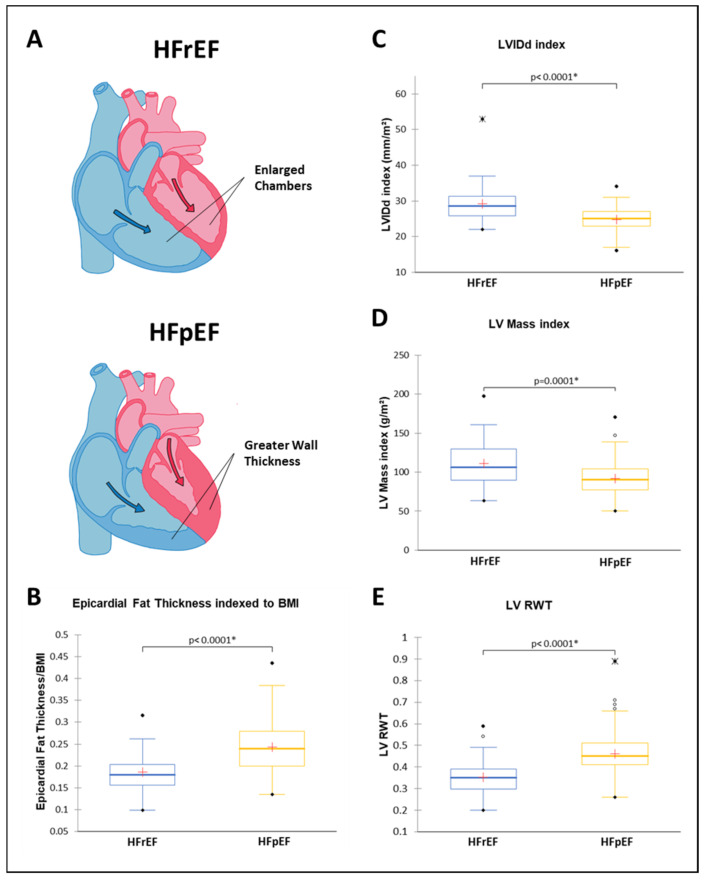
(**A**) Diagrammatic illustration comparing the structural differences in heart morphology between HFrEF and HFpEF. Arrows represent blood flow during diastole. Blue shading signifies the right heart receiving deoxygenated blood and pink shading signifies the left heart receiving oxygenated blood. (**B**) Box plots of epicardial fat thickness measured by echocardiography and indexed to BMI stratified to HFrEF (*n* = 40) and HFpEF (*N* = 141). (**C**) Box plots of LVIDd Index measured by echocardiography stratified to HFrEF (*n* = 40) and HFpEF (*N* = 140). (**D**) Box plots of LV Mass Index measured by echocardiography stratified to HFrEF (*n* = 40) and HFpEF (*N* = 138). (**E**) Box plots of LV RWT measured by echocardiography stratified to HFrEF (*n* = 40) and HFpEF (*N* = 141). BMI, body mass index; HFpEF, heart failure with preserved ejection fraction; HFrEF, heart failure with reserved ejection fraction; LV, left ventricular; RWT, relative wall thickness. * *p* < 0.05 indicating a significant difference between HFrEF and HFpEF groups.

The echocardiographic findings presented in Table 2 demonstrate that HFpEF patients had thicker left ventricular wall dimensions as evidenced by significantly (*p* < 0.05) greater mean values for interventricular septum thickness in end-diastole (IVSd), left ventricular posterior wall thickness in end-diastole (LVPWd), and left ventricular relative wall thickness (LV RWT). HFrEF patients had larger hearts with more mass, larger cardiac chambers, and higher cardiac chamber volumes which was reflected in significantly (*p* < 0.05) greater mean values for left ventricular internal diameter in end-diastole (LVIDd), left ventricular internal diameter in end-systole (LVIDs), left ventricular (LV) mass index, left ventricular end-diastolic volume (LV EDV) index, left ventricular end-systolic volume (LV ESV) index, left ventricular outflow tract (LVOT) diameter, left atrium size, and left atrial (LA) volume index. These phenotypic differences between HFpEF and HFrEF are displayed in Figure 2A,C–E. HFrEF patients also had significantly (*p* < 0.05) larger mean values for right ventricular diameter (RVd A4C) and right atrial volume. HFrEF patients had decreased right ventricular function compared with HFpEF patients with significantly (*p* < 0.05) lower mean values for right ventricular (RV) S’ and Tricuspid annular plane systolic excursion (TAPSE) along with a significantly (*p* < 0.05) higher right atrial pressure. Other notable findings include a significantly longer deceleration time and a significantly faster septal e’ velocity in HFpEF patients. The mean values for mitral valve (MV) peak E, MV peak A, MV E/A ratio, lateral e’, average E/e’ ratio, tricuspid regurgitation max velocity, and pulmonary artery systolic pressure (PASP) were not significantly different between HFrEF and HFpEF.

Epicardial fat thickness was significantly (*p* < 0.0001) greater in HFpEF patients with a mean of 6.7 ± 1.6 mm compared with a mean of 5.1 ± 1.0 mm in HFrEF patients. Increased epicardial fat content has been shown to be associated with obesity and BMI [11,28,29] and in order to adjust for this relationship, epicardial fat thickness was divided by BMI [29]. After adjustment for BMI, epicardial fat thickness was still significantly (*p* < 0.0001) greater in HFpEF compared with HFrEF (Figure 2B). There were no significant differences in epicardial fat thickness regardless of BMI when comparing mild diastolic dysfunction with moderate/severe diastolic dysfunction within the HFpEF patients. Epicardial fat thickness/BMI was nearly identical between these two subsets of HFpEF patients with measurements of 0.24 ± 0.06 and a *p*-value of 0.896.

**Figure 3:**
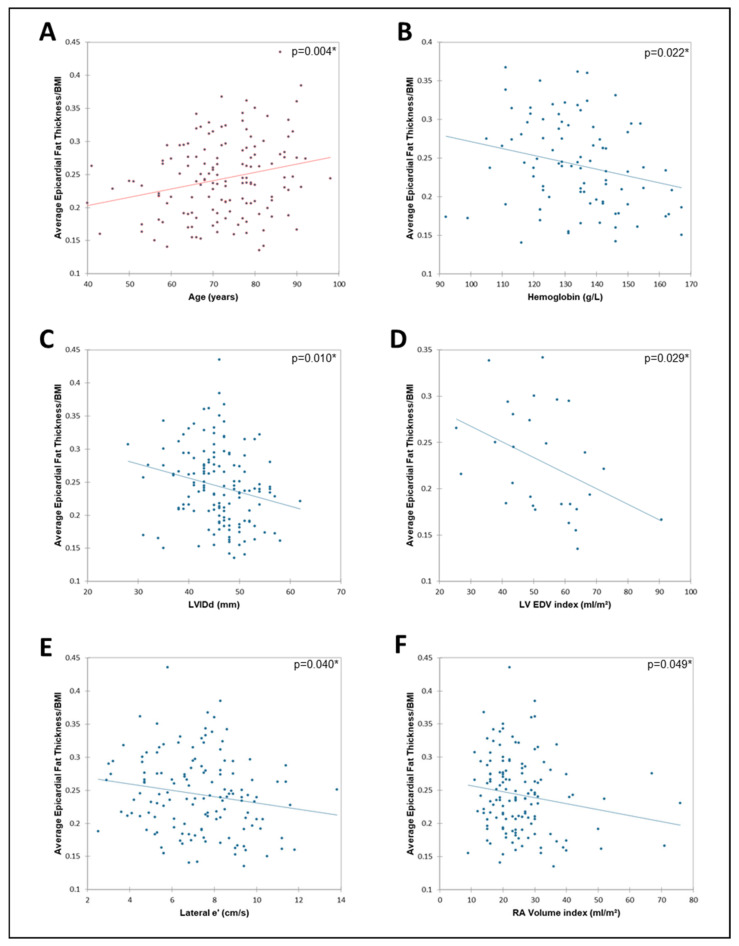
In HFpEF, the linear association of epicardial fat thickness indexed to BMI with (**A**) age (*n* = 141), (**B**) hemoglobin (*n* = 90), (**C**) LVIDd (*n* = 141), (**D**) LV EDV index (*n* = 28), (**E**) lateral e’ (*n* = 139), and (**F**) RA Volume index (*n* = 134). BMI, body mass index; EDV, end-diastolic volume; HFpEF, heart failure with preserved ejection fraction; LV, left ventricular; LVIDd, left ventricular internal diameter end diastole; RA, right atrial volume index. * p < 0.05 indicating a significant linear correlation. Red shading signifies a positive association and blue shading signifies a negative association.

Several significant correlations were identified between epicardial fat thickness and a number of other variables (Table 3 and Table 4). Since the associations may or may not be parametric, both Pearson’s correlation tests and Spearman’s correlation tests were employed. In HFpEF, a significant (*p* < 0.05) positive linear relationship was found between epicardial fat and age as well as significant (*p* < 0.05) negative correlations with hemoglobin (Hgb), LVIDd, LVIDs, LV EDV index, lateral e’, septal e’, and RA volume index (Figure 3A–F). In HFrEF, epicardial fat was significantly (*p* < 0.05) positively correlated with LVIDd index and negatively correlated with left atrium size and right atrial pressure. The relationships were similar when the data were analyzed for non-linear relationships (Table 4). There was no significant correlation between epicardial fat and LV ejection fraction. Only in HFpEF, epicardial fat thickness/BMI was positively correlated with age and negatively correlated with Hgb, LVIDd, LVIDs, LV EDV index, and lateral e’. In HFrEF, epicardial fat thickness was positively correlated with septal e’ and negatively correlated with left atrium size and RA pressure in non-parametric statistical testing. No other significant associations were found for the epicardial fat. The relationship between epicardial fat and hemoglobin (Hgb) in HFpEF was explored further. There was a highly significant (r = −0.272; *p* = 0.006) relationship between age and hemoglobin (Hgb). Multivariate (multilinear) analysis including age, epicardial fat, and Hgb found that after adjusting for age, there was no significant (*p* = 0.12) relationship between epicardial fat and Hgb. In contrast to other variables, there remained a highly significant (*p* = 0.0025) relationship between epicardial fat and RA volume index even after adjusting for age. 

## 4. Discussion

This study had several key significant findings. Epicardial fat was greater in patients with HFpEF than patients with HFrEF. This relationship was re-evaluated after taking into account that epicardial fat is positively associated with general body adiposity as measured by BMI [11,28,29]. After accounting for this confounder, epicardial fat thickness was significantly greater in patients with HFpEF or diastolic dysfunction compared with patients with HFrEF. Importantly, there are distinctly different relationships between epicardial fat and echocardiographic dimensions as well as other factors in HFpEF compared with HFrEF. In HFpEF but not HFrEF, epicardial fat correlates significantly with age, LVIDd, LVIDs, LV EDV index, RA volume index, lateral e’, and septal e’.

A significant negative correlation was observed between epicardial fat and left ventricular internal diameters, such that the greater the amount of epicardial fat, the smaller the LVIDd, LVIDs, and LV EDV index. This novel relationship was unique to HFpEF compared with HFrEF. There is limited previous data on these relationships and inconsistent data on the association between epicardial fat and cardiac structure [21]. We contend that it is reasonable to suggest that epicardial fat can play a role in mechanically restricting cardiac expansion and preventing ventricular distension [20,30,31]. Our findings are consistent with the concept that epicardial fat plays a role in LV stiffening and diastolic dysfunction, such that increasing epicardial fat will progressively impair diastolic filling leading to a lower LV EDV index. We found no correlation of epicardial fat with LV ejection fraction in patients with HFrEF.

We found that increased epicardial fat was associated with a smaller RA volume index in HFpEF. Epicardial fat occurs in greater amounts in areas adjacent to the right atrium [11]. We believe that mechanisms analogous to the ones discussed above for the left ventricle are likely responsible for this negative association, namely, mechanical constriction and increased fibrosis of the myocardium caused by increasing epicardial fat. This finding is consistent with the proposed role of epicardial fat in driving atrial remodeling and atrial fibrillation burden [32] as well as the proposed role of HFpEF in the pathogenesis of atrial fibrillation [33].

Together, lateral e’ and septal e’ are one of the four recommended variables utilized to determine if LV diastolic function is normal or abnormal [22]. In our study, in HFpEF, epicardial fat thickness was negatively correlated with lateral e’ and septal e’. Although, lateral e’ and septal e’ are not directly utilized for grading LV diastolic dysfunction, they can be used as a proxy for quantifying diastolic function in this case with lower values being indicative of worsening LV diastolic function. Based on these findings, an increase in epicardial fat is associated with worse echocardiographic markers of diastolic dysfunction in patients with HFpEF. This conclusion is consistent with other studies [14,18,21,34].

It is reasonable to hypothesize that disease pathophysiology and perhaps genetic factors [35] may influence the dysregulation of epicardial fat accumulation in HFpEF compared with HFrEF. It has been proposed that the increased energy demands of the heart in HFrEF may be responsible for the consumption of epicardial fat and the subsequent thinning of epicardial adipose tissue in HFrEF compared with HFpEF [36]. Alternatively, epicardial fat may play a role in the pathophysiology of HFpEF. Adipose tissue is maintained in its location by a fibrous tissue scaffold which can pack fat onto the surface of the heart adding a mass of resistance to the diastolic expansion of the heart [20]. Epicardial fat-induced reduction in coronary blood flow may also be a contributing factor [37]. Increased LV stiffness in HFpEF can be attributed to myocardial fibrosis caused by an increased deposition of collagen as a result of enhanced synthesis and/or diminished degradation caused by the dysregulation of relevant enzymes [5,38]. There is a robust positive correlation between epicardial fat volume and the amount of fibrosis as indicated by myocardial extracellular volume, particularly in HFpEF patients [17,39]. Other factors may contribute independently to the pathogenesis of HFpEF and epicardial fat accumulation. However, hypertension, which can be associated with increased epicardial fat volume [40], did not differ in prevalence between HFpEF and HFrEF in our study.

Epicardial fat is a metabolically active tissue with a distinct gene expression profile compared with other types of adipose tissue [9,11,12,13]. Epicardial fat is a focal source of pro-inflammatory and pro-fibrotic cytokines which can enter the microcirculation shared with myocardium to induce myocardial fibrosis [17,19,31]. Another proposed mechanism that implicates epicardial fat in diastolic dysfunction is the hypothesized direct infiltration of epicardial fat into the myocardium as intramyocardial fat, directly affecting LV stiffness and local inflammation [20,41,42]. Lastly, the mass effect of expanded epicardial adipose tissue in a fixed (pericardial) space has been postulated to impose a mechanical ‘constrictive’ effect on the heart, impairing diastolic filling and influencing ventricular remodeling [16,20,31,43]. Epicardial fat can be reduced by exercise and by bariatric surgery in the morbidly obese [44], providing an avenue by which one can investigate a causative link between epicardial fat and HFpEF [45]. Taken together, our data support the concept that epicardial fat thickness and total epicardial fat volume is generally the highest in patients with HFpEF and the lowest in patients with HFrEF with healthy controls falling somewhere in between [19,46].

Our findings suggest that the prototypical heart with HFpEF has a smaller mass and lower ventricular volumes, thicker wall dimensions, and smaller chamber dimensions when compared with the prototypical heart with HFrEF. Similarly, left atrium size, LA volume, RVd A4C, and RA volumes were smaller in HFpEF than HFrEF. With regards to wall thickness, significantly greater values for IVSd, LVPWd, and LV RWT were identified in HFpEF than HFrEF. While these findings can be attributed to concentric remodeling and concentric hypertrophy, a morphological marker of hypertensive heart disease [47], hypertension prevalence was not different in HFpEF compared with HFrEF in our study.

With regards to LV mass, volumes, and dimensions, LVIDd, LVIDd, LVIDs, LV Mass index, LV EDV index, and LV ESV index were all found to be significantly greater in HFrEF, which is in agreement with previous reports [48,49]. Right ventricular systolic dysfunction, as measured by TAPSE and RV S’, was significantly worse in HFrEF which is in consensus with the literature [48,50]. Notably, right ventricular systolic dysfunction in HFpEF is a marker of increased morbidity and mortality [51,52].

Additionally, epicardial fat was found to be negatively correlated with hemoglobin in HFpEF which is a novel finding. The explanation for this finding is mostly likely due to the association of increasing age with increasing epicardial fat and decreasing hemoglobin. It is unlikely that epicardial fat inhibits erythropoiesis. Interestingly, we have previously outlined a role for HIF-1 alpha in HFpEF [7]. HIF-1 alpha can exert an antagonistic effect on HIP-2 alpha which regulates erythropoietin synthesis. The HIF pathway is present in most cells in the body and is involved in cellular adaptation to reduced oxygen and can be involved in erythropoiesis [53].

In our HFpEF cohort, we observed a significant positive correlation between epicardial fat and age consistent with the findings of some other studies [54,55]. HFpEF and diastolic dysfunction are associated with older age and longitudinal studies show a gradual worsening of diastolic function with normal aging. Taken together, one can speculate that the risk of HFpEF and diastolic dysfunction is a function of increasing epicardial fat with age.

## 5. Limitations

Several limitations of the study warrant consideration. Firstly, epicardial fat was measured in one dimension and location but total epicardial fat mass was not determined, such as by MRI. However, epicardial fat thickness as measured by transthoracic echocardiography has been shown to be a suitable proxy measurement for total epicardial fat volume as measured by CT and MRI, so it is reasonable to extrapolate our findings to total epicardial fat volume [27]. Secondly, this was a retrospective study which has its limitations in defining the composition of the study populations and therefore important subgroups, such as females and individuals with atrial fibrillation, may be underrepresented compared with epidemiological studies. Furthermore, retrospective studies are not able to make conclusions about causation and we are unable to determine if increased epicardial fat is a cause or consequence of HFpEF. We can only speculate that increased epicardial fat drives inflammatory processes that ultimately influence the pathogenesis of HFpEF. Additionally, the sample size used in this research study was too small to compare epicardial fat thickness between identified subtypes of HFpEF elucidated using machine learning [56]. Lastly, we did not perform an in-depth multivariate analysis of diastolic function.

It would also be interesting to know at what level of epicardial fat thickness there would be an anticipated effect on diastolic dysfunction. However, this type of analysis would require a control group to construct different kinds of statistical evaluations such as under the curve (AUC) analysis. This can be potentially explored in future studies.

It is useful to consider the limitations of the current assessment of diastolic function by echocardiography. Diastolic LV function (and dysfunction) is best quantified by the time constant of isovolumic relaxation (tau) calculated from measurement of LV volume and instantaneous recording from a high-fidelity LV pressure tracing during the period of isovolumic relaxation. Furthermore, there should be an intervention to change LV volume to determine its impact on the LV pressure/LV volume relationship in order to calculate the time constants of isovolumic relaxation. The echocardiogram is an indirect approximation of diastolic function/dysfunction. Our data must be viewed within the constraints of the echocardiographic assessment of diastolic function.

## 6. Conclusions

There are several key findings of the present study (Figure 2). Patients with HFpEF had a higher burden of epicardial fat compared with patients with HFrEF. In HFpEF, the amount of epicardial fat was directly associated with smaller LV internal diameters, LV EDV indexes, lateral e’, and septal e’. Hearts with HFpEF were significantly smaller in size, mass, and volume but had thicker wall dimensions than hearts with HFrEF. These findings highlight the differences between HFpEF and HFrEF and suggest clinical trials to reduce epicardial fat to determine its role in the pathogenesis and clinical course of HFpEF. Intensive weight reduction with lifestyle, pharmacological, and/or surgical interventions can potentially be recommended as future therapeutic avenues for HFpEF.

## Figures and Tables

**Table 1:** Main Characteristics of the Study Population (HFrEF vs. HFpEF).

	*n*	HFrEF Patients	*N*	HFpEF Patients	*p*-Value
# of Patients	40	40 (22.1)	141	141 (77.9)	-
Age (years)	40	71.3 ± 12.6	141	72.2 ± 11.2	0.841
Sex	40		141		0.131
Male		28 (70.0)		80 (56.7)	
Female		12 (30.0)		61 (43.3)	
Height (metres)	40	1.73 ± 0.11	141	1.67 ± 0.11 *	0.013
Weight (kilograms)	40	84.4 ± 19.8	141	78.4 ± 17.4	0.055
BMI (kg/m^2^)	40	28.3 ± 5.5	141	27.9 ± 5.1	0.316
BSA (m^2^)	40	1.98 ± 0.27	141	1.87 ± 0.24 *	0.022
Hypertension	40	32 (80.0)	141	122 (86.5)	0.307
Current Smoker	40	5 (12.5)	141	6 (4.3)	0.054
Past Smoking History	40	23 (57.5)	141	59 (41.8)	0.079
Diabetes Mellitus	40	15 (37.5)	141	45 (31.9)	0.508
Dyslipidemia	40	31 (77.5)	141	105 (74.5)	0.695
Chronic Kidney Disease ^a^	40	20 (50.0)	141	46 (32.6) *	0.044
Coronary Artery Disease	40	23 (57.5)	141	53 (37.6) *	0.024
Previous Myocardial Infarct	40	12 (30.0)	141	18 (12.8) *	0.010
Atrial Fibrillation	40	10 (25.0)	141	25 (17.7)	0.304
Medications					
ACEi/ARB use	40	35 (87.5)	141	100 (70.9) *	0.034
CCB use	40	10 (25.0)	141	56 (39.7)	0.088
Beta Blocker use	40	32 (80.0)	141	76 (53.9) *	0.003
Thiazide Diuretic use	40	8 (20.0)	141	50 (35.5)	0.064
Loop Diuretic use	40	26 (65.0)	141	21 (14.9) *	<0.0001
Vasodilator use	40	8 (20.0)	141	12 (8.5) *	0.041
Statin use	40	28 (70.0)	141	93 (66.0)	0.632
Cholesterol Absorption Inhibitor use	40	3 (7.5)	141	2 (1.4) *	0.038
SGLT2 Inhibitor use	40	7 (17.5)	141	6 (4.3) *	0.004
MRA use	40	15 (37.5)	141	15 (10.6) *	<0.0001
Oral Hypoglycemic Agent use	40	11 (27.5)	141	26 (18.4)	0.210
Clinical and Laboratory					
Creatinine (μmol/L)	39	118 ± 41	126	103 ± 65 *	0.004
eGFR (ml/min)	38	57 ± 21	126	64 ± 22	0.091
Hemoglobin (g/L)	34	128 ± 18	90	133 ± 15	0.113
BNP (ng/L)	21	489 ± 641	41	172 ± 264 *	0.002
BNP Prohormone (ng/L)	6	1935 ± 1049	4	671 ± 375	0.067
Systolic Blood Pressure (mmHg)	39	135 ± 19	138	147 ± 22 *	0.001
Diastolic Blood Pressure (mmHg)	39	72 ± 14	138	74 ± 11	0.068
Heart Rate (beats/min)	21	76 ± 18	193	69 ± 13 *	0.050

Values are mean ± standard deviation or *n*(%). ACEi, angiotensin-converting enzyme inhibitor; ARB, angiotensin receptor blocker; BMI, body mass index; BNP, brain natriuretic peptide; BSA, body surface area; CCB, calcium channel blocker; eGFR, estimated glomerular filtration rate; HFpEF, heart failure with preserved ejection fraction; HFrEF, heart failure with reduced ejection fraction; MRA, mineralocorticoid receptor antagonist; SGLT2 Inhibitor; sodium-glucose cotransporter-2 inhibitor. ^a^ Chronic kidney disease defined as eGFR ≤ 60 mL/min. * *p* **<** 0.05 vs. HFrEF.

**Table 2:** Echocardiographic Characteristics of the Study Population.

	*n*	HFrEF Patients	*N*	HFpEF Patients	*p*-Value
**Epicardial Fat**					
Epicardial Fat Thickness (mm)	40	5.1 ± 1.0	141	6.7 ± 1.6 *	<0.0001
Epicardial Fat Thickness/BMI	40	0.19 ± 0.04	141	0.24 ± 0.06 *	<0.0001
**Left Ventricle**					
IVSd (mm)	40	9.7 ± 1.5	141	10.7 ± 1.7 *	0.0004
LVPWd (mm)	40	9.8 ± 1.4	141	10.3 ± 1.5 *	0.009
LVIDd (mm)	40	56.7 ± 9.1	141	45.9 ± 5.9 *	<0.0001
LVIDd index (mm/m^2^)	40	29.1 ± 5.4	140	24.8 ± 3.2 *	<0.0001
LVIDs (mm)	38	45.9 ± 9.3	137	29.9 ± 5.2 *	<0.0001
LV EF (%)	39	34.7 ± 7.7	141	60.1 ± 5.3 *	<0.0001
LV Mass Index (g/m^2^)	40	111.1 ± 29.6	138	91.6 ± 21.2 *	0.0001
LV RWT	40	0.35 ± 0.08	141	0.46 ± 0.10 *	<0.0001
LV EDV Index (ml/m^2^)	29	81.0 ± 33.4	28	53.0 ± 14.1 *	<0.0001
LV ESV Index (ml/m^2^)	28	54.9 ± 27.8	28	22.8 ± 7.4 *	<0.0001
LVOT Diameter (mm)	34	21.5 ± 1.9	136	20.6 ± 1.9 *	0.012
**LV Diastolic Function**					
MV Peak E (cm/s)	39	77.9 ± 25.3	141	76.8 ± 25.0	0.749
MV Peak A (cm/s)	27	79.1 ± 27.3	136	82.5 ± 26.1	0.467
Deceleration Time (msec)	37	186 ± 51	137	247 ± 74 *	<0.0001
MV E/A ratio	27	1.03 ± 0.54	136	0.98 ± 0.47	0.975
Lateral e’ (cm/s)	38	7.2 ± 3.0	139	7.3 ± 2.1	0.396
Septal e’ (cm/s)	39	4.7 ± 1.5	139	5.5 ± 1.5 *	0.008
Average E/e’ Ratio	38	14.0 ± 6.1	138	12.7 ± 5.3	0.336
**Left Atrium**					
Left Atrium (mm)	38	42.8 ± 8.4	137	38.8 ± 6.2 *	0.004
LA Volume Index (Biplane) (ml/m^2^)	39	45.8 ± 12.3	141	39.7 ± 12.8 *	0.002
**Right Ventricle**					
RVd A4C (mm)	37	37.5 ± 6.8	139	34.7 ± 5.1 *	0.028
RV S’ (cm/s)	25	10.9 ± 2.2	99	12.6 ± 2.9 *	0.006
TAPSE (mm)	37	19.4 ± 3.2	129	22.2 ± 4.2 *	< 0.0001
**Right Atrium**					
RA Volume Index (ml/m^2^)	36	31.7 ± 16.6	134	25.2 ± 10.7 *	0.007
**Tricuspid Valve and PA/RV Systolic Pressure**					
TR Max Velocity (m/s)	26	2.65 ± 0.34	96	2.54 ± 0.36	0.128
RA Pressure (mmHg)	37	4.4 ± 2.3	132	3.5 ± 1.7 *	0.003
PASP (mmHg)	25	32.4 ± 7.8	93	29.6 ± 8.3	0.053

Values are mean ± standard deviation. EF, ejection fraction; EDV, end-diastolic volume; ESV, end-systolic volume; HFpEF, heart failure with preserved ejection fraction; HFrEF, heart failure with reduced ejection fraction; IVSd, interventricular septum thickness end diastole; LA, left atrial; LV, left ventricular; LVIDd, left ventricular internal diameter end diastole; LVIDs, left ventricular internal diameter end systole; LVOT, left ventricular outflow tract; LVPWd, left ventricular posterior wall end diastole; MV, mitral valve; PA, pulmonary artery; PASP, pulmonary artery systolic pressure; RWT, relative wall thickness; RA, right atrial; RV, right ventricular; RVd A4C; right ventricular diameter apical 4 chamber view; TAPSE, tricuspid annular plane systolic excursion; TR, tricuspid regurgitation.* *p* < 0.05 vs. HFrEF.

**Table 3:** Significant Pearson’s Correlations (Linear relationship) with Epicardial Fat Thickness/BMI.

	Epicardial Fat Thickness/BMI					
	HFrEF Patients (*n* = 40)			HFpEF Patients (*N* = 141)		
	Sample Size (*n*)	R	*p*-Value	Sample Size (*N*)	R	*p*-Value
Age (years)	40	−0.040	0.808	141	0.242 *	0.004
Weight (kilograms)	40	−0.428 *	0.006	141	−0.320 *	0.0001
BMI (kg/m^2^)	40	−0.637 *	<0.0001	141	−0.342 *	<0.0001
BSA (m^2^)	40	−0.281	0.079	141	−0.268 *	0.001
Hemoglobin (g/L)	34	0.086	0.631	90	−0.241 *	0.022
LVIDd (mm)	40	0.049	0.762	141	−0.217 *	0.010
LVIDd Index (mm/m^2^)	40	0.414 *	0.008	140	0.033	0.697
LVIDs (mm)	38	−0.048	0.774	137	−0.205 *	0.016
LV EDV Index (ml/m^2^)	29	0.114	0.555	28	−0.413 *	0.029
Lateral e’ (cm/s)	38	0.068	0.683	139	−0.174 *	0.040
Septal e’ (cm/s)	39	0.308	0.057	139	−0.171 *	0.044
Left Atrium (mm)	38	−0.365 *	0.024	137	−0.049	0.570
RA Volume index (ml/m^2^)	36	−0.089	0.607	134	−0.170 *	0.049
RA Pressure (mmHg)	37	−0.399 *	0.014	132	−0.158	0.070

BMI, body mass index; BSA, body surface area; EDV, end-diastolic volume; HFpEF, heart failure with preserved ejection fraction; HFrEF, heart failure with reduced ejection fraction; LV, left ventricular; LVIDd, left ventricular internal diameter end diastole; LVIDs, left ventricular internal diameter end systole; RA, right atrial. * *p* < 0.05 indicating a significant correlation.

**Table 4:** Significant Spearman’s Correlations (Non-linear relationship) with Epicardial Fat Thickness/BMI.

	Epicardial Fat Thickness/BMI (*n* = 181)					
	HFrEF Patients (*n* = 40)			HFpEF Patients (*N* = 141)		
	Sample Size (*n*)	R	*p*-Value	Sample Size (*N*)	R	*p*-Value
Age (years)	40	−0.188	0.244	141	0.207 *	0.014
Weight (kilograms)	40	−0.297	0.063	141	−0.351 *	<0.0001
BMI (kg/m^2^)	40	−0.507 *	0.001	141	−0.348 *	<0.0001
BSA (m^2^)	40	−0.132	0.414	141	−0.280 *	0.001
Hemoglobin (g/L)	34	0.157	0.374	90	−0.266 *	0.012
LVIDd (mm)	40	0.100	0.539	141	−0.269 *	0.001
LVIDs (mm)	38	−0.079	0.637	137	−0.235 *	0.006
LV EDV Index (ml/m^2^)	29	−0.093	0.629	28	−0.456 *	0.015
Lateral e’ (cm/s)	38	0.055	0.740	139	−0.178 *	0.037
Septal e’ (cm/s)	39	0.361 *	0.025	139	−0.153	0.072
Left Atrium (mm)	38	−0.357 *	0.028	137	−0.056	0.514
RA Pressure (mmHg)	37	−0.393 *	0.017	132	−0.173 *	0.047

BMI, body mass index; BSA, body surface area; EDV, end-diastolic volume; HFpEF, heart failure with preserved ejection fraction; HFrEF, heart failure with reduced ejection fraction; LV, Left ventricular; LVIDd, left ventricular internal diameter end diastole; LVIDs, left ventricular internal diameter end systole; RA, right atrial. * *p* < 0.05 indicating a significant correlation.

## Data Availability

The original contributions presented in the study are included in the article, further inquiries can be directed to the corresponding authors.

## References

[1] Steinberg B.A., Zhao X., Heidenreich P.A., Peterson E.D., Bhatt D.L., Cannon C.P., Hernandez A.F., Fonarow G.C. (2012). Trends in Patients Hospitalized with Heart Failure and Preserved Left Ventricular Ejection Fraction: Prevalence, Therapies, and Outcomes. Circulation.

[2] Anker S.D., Butler J., Filippatos G., Ferreira J.P., Bocchi E., Bohm M., Brunner-La Rocca H.-P., Choi D.-J., Chopra V., Chuquiure-Valenzuela E. (2021). Empagliflozin in Heart Failure with a Preserved Ejection Fraction. N. Engl. J. Med..

[3] Solomon S., McMurray J.J.V., Brian Claggett B., de Boer R., DeMets D., Hernandez A., Inzucchi S., Kosiborod M., Lam C., Martinez F. (2022). Dapagliflozin in Heart Failure with Mildly Reduced or Preserved Ejection Fraction. N. Engl. J. Med..

[4] Borbely A., van der Velden J., Papp Z., Bronzwaer J.G.F., Edes I., Stienen G.J.M., Paulus W.J. (2005). Cardiomyocyte Stiffness in Diastolic Heart Failure. Circulation.

[5] Weber K.T., Brilla C.G., Janicki J.S. (1993). Myocardial Fibrosis: Functional Significance and Regulatory Factors. Cardiovasc. Res..

[6] Mishra S., Kass D.A. (2021). Cellular and Molecular Pathobiology of Heart Failure with Preserved Ejection Fraction. Nat. Rev. Cardiol..

[7] Warbrick I., Rabkin S.W. (2019). Hypoxia-Inducible Factor 1-Alpha (HIF-1α) as a Factor Mediating the Relationship between Obesity and Heart Failure with Preserved Ejection Fraction. Obes. Rev..

[8] Borlaug B.A. (2014). The Pathophysiology of Heart Failure with Preserved Ejection Fraction. Nat. Rev. Cardiol..

[9] Ouwens D.M., Sell H., Greulich S., Eckel J. (2010). The Role of Epicardial and Perivascular Adipose Tissue in the Pathophysiology of Cardiovascular Disease. J. Cell. Mol. Med..

[10] Iacobellis G., Barbaro G. (2008). The Double Role of Epicardial Adipose Tissue as Pro- and Anti-Inflammatory Organ. Horm. Metab. Res..

[11] Rabkin S.W. (2007). Epicardial Fat: Properties, Function and Relationship to Obesity. Obes. Rev..

[12] Agra R.M., Teijeira-Fernández E., Pascual-Figal D., Sánchez-Más J., Fernández-Trasancos Á., González-Juanatey J.R., Eiras S. (2014). Adiponectin and P53 MRNA in Epicardial and Subcutaneous Fat from Heart Failure Patients. Eur. J. Clin. Investig..

[13] Yim J., Rabkin S.W. (2017). Differences in Gene Expression and Gene Associations in Epicardial Fat Compared to Subcutaneous Fat. Horm. Metab. Res..

[14] Lin H.H., Lee J.K., Yang C.Y., Lien Y.C., Huang J.W., Wu C.K. (2013). Accumulation of Epicardial Fat Rather than Visceral Fat Is an Independent Risk Factor for Left Ventricular Diastolic Dysfunction in Patients Undergoing Peritoneal Dialysis. Cardiovasc. Diabetol..

[15] van Woerden G., Gorter T.M., Westenbrink B.D., Willems T.P., van Veldhuisen D.J., Rienstra M. (2018). Epicardial Fat in Heart Failure Patients with Mid-Range and Preserved Ejection Fraction. Eur. J. Heart Fail..

[16] Koepp K.E., Obokata M., Reddy Y.N.V., Olson T.P., Borlaug B.A. (2020). Hemodynamic and Functional Impact of Epicardial Adipose Tissue in Heart Failure With Preserved Ejection Fraction. JACC Hear. Fail..

[17] Tromp J., Bryant J.A., Jin X., van Woerden G., Asali S., Yiying H., Liew O.W., Ching J.C.P., Jaufeerally F., Loh S.Y. (2021). Epicardial Fat in Heart Failure with Reduced versus Preserved Ejection Fraction. Eur. J. Hear. Fail..

[18] Ma W., Zhang B., Yang Y., Qi L., Zhou J., Li M., Jia J., Zhang Y., Yong H. (2021). Association of Epicardial Fat Thickness with Left Ventricular Diastolic Function Parameters in a Community Population. BMC Cardiovasc. Disord..

[19] Pugliese N.R., Paneni F., Mazzola M., De Biase N., Del Punta L., Gargani L., Mengozzi A., Virdis A., Nesti L., Taddei S. (2021). Impact of Epicardial Adipose Tissue on Cardiovascular Haemodynamics, Metabolic Profile, and Prognosis in Heart Failure. Eur. J. Hear. Fail..

[20] Rabkin S.W. (2017). Is Reduction in Coronary Blood Flow the Mechanism by Which Epicardial Fat Produces Left Ventricular Diastolic Dysfunction?. Can. J. Cardiol..

[21] Nerlekar N., Muthalaly R.G., Wong N., Thakur U., Wong D.T.L., Brown A.J., Marwick T.H. (2018). Association of Volumetric Epicardial Adipose Tissue Quantification and Cardiac Structure and Function. J. Am. Hear. Assoc..

[22] Nagueh S.F., Smiseth O.A., Appleton C.P., Byrd B.F., Dokainish H., Edvardsen T., Flachskampf F.A., Gillebert T.C., Klein A.L., Lancellotti P. (2016). Recommendations for the Evaluation of Left Ventricular Diastolic Function by Echocardiography: An Update from the American Society of Echocardiography and the European Association of Cardiovascular Imaging. Eur. Hear. J. Cardiovasc. Imaging.

[23] Zoghbi W.A., Adams D., Bonow R.O., Enriquez-Sarano M., Foster E., Grayburn P.A., Hahn R.T., Han Y., Hung J., Lang R.M. (2017). Recommendations for noninvasive evaluation of native valvular regurgitation. J. Am. Soc. Echocardiogr..

[24] Baumgartner H., Hung J., Bermejo J., Chambers J.B., Edvardsen T., Goldstein S., Lancellotti P., LeFevre M., Miller F., Otto C.M. (2017). Recommendations on the echocardiographic assessment of Aortic Valve Stenosis: A focused update from the European Association of Cardiovascular Imaging and the American Society of Echocardiography. J. Am. Soc. Echocardiogr..

[25] Cetin M., Kocaman S.A., Durakoglugil M.E., Erdogan T., Ergul E., Dogan S., Canga A. (2013). Effect of Epicardial Adipose Tissue on Diastolic Functions and Left Atrial Dimension in Untreated Hypertensive Patients with Normal Systolic Function. J. Cardiol..

[26] Iacobellis G., Willens H.J. (2009). Echocardiographic Epicardial Fat: A Review of Research and Clinical Applications. J. Am. Soc. Echocardiogr..

[27] Hirata Y., Yamada H., Kusunose K., Iwase T., Nishio S., Hayashi S., Bando M., Amano R., Yamaguchi K., Soeki T. (2015). Clinical Utility of Measuring Epicardial Adipose Tissue Thickness with Echocardiography Using a High-Frequency Linear Probe in Patients with Coronary Artery Disease. J. Am. Soc. Echocardiogr..

[28] Rabkin S.W. (2014). The Relationship between Epicardial Fat and Indices of Obesity and the Metabolic Syndrome: A Systematic Review and Meta-Analysis. Metab. Syndr. Relat. Disord..

[29] Aitken-Buck H.M., Moharram M., Babakr A.A., Reijers R., Van Hout I., Fomison-Nurse I.C., Sugunesegran R., Bhagwat K., Davis P.J., Bunton R.W. (2019). Relationship between Epicardial Adipose Tissue Thickness and Epicardial Adipocyte Size with Increasing Body Mass Index. Adipocyte.

[30] Koepp K.E., Borlaug B.A. (2020). Reply: Epicardial Adipose Tissue in Heart Failure With Preserved Ejection Fraction: Matter of Preference or Evidence?. JACC Hear. Fail..

[31] Salvatore T., Galiero R., Caturano A., Vetrano E., Rinaldi L., Coviello F., Di Martino A., Albanese G., Colantuoni S., Medicamento G. (2022). Dysregulated Epicardial Adipose Tissue as a Risk Factor and Potential Therapeutic Target of Heart Failure with Preserved Ejection Fraction in Diabetes. Biomolecules.

[32] Al Chekakie M.O., Akar J.G. (2012). Epicardial Fat and Atrial Fibrillation: A Review. J. Atr. Fibrillation.

[33] Rabkin S.W., Nourai H. (2022). Atrial Fibrillation in Heart Failure with Preserved Ejection Fraction. J. Atr. Fibrillation.

[34] Maimaituxun G., Yamada H., Fukuda D., Yagi S., Kusunose K., Hirata Y., Nishio S., Soeki T., Masuzaki H., Sata M. (2020). Association of Local Epicardial Adipose Tissue Depots and Left Ventricular Diastolic Performance in Patients with Preserved Left Ventricular Ejection Fraction. Circ. J..

[35] Granér M., Seppälä-Lindroos A., Rissanen A., Hakkarainen A., Lundbom N., Kaprio J., Nieminen M.S., Pietiläinen K.H. (2012). Epicardial Fat, Cardiac Dimensions, and Low-Grade Inflammation in Young Adult Monozygotic Twins Discordant for Obesity. Am. J. Cardiol..

[36] Tromp J., Packer M., Lam C.S. (2021). The Diverging Role of Epicardial Adipose Tissue in Heart Failure with Reduced and Preserved Ejection Fraction: Not All Fat Is Created Equal. Eur. J. Heart Fail..

[37] Nakanishi K., Fukuda S., Tanaka A., Otsuka K., Taguchi H., Shimada K. (2017). Relationships Between Periventricular Epicardial Adipose Tissue Accumulation, Coronary Microcirculation, and Left Ventricular Diastolic Dysfunction. Can. J. Cardiol..

[38] Chirinos J.A., Akers S.R., Trieu L., Ischiropoulos H., Doulias P.-T., Tariq A., Vasim I., Koppula M.R., Syed A.A., Soto-Calderon H. (2016). Heart Failure, Left Ventricular Remodeling, and Circulating Nitric Oxide Metabolites. J. Am. Heart Assoc..

[39] Wu C.K., Tsai H.Y., Su M.Y.M., Wu Y.F., Hwang J.J., Lin J.L., Lin L.Y., Chen J.J. (2017). Evolutional Change in Epicardial Fat and Its Correlation with Myocardial Diffuse Fibrosis in Heart Failure Patients. J. Clin. Lipidol..

[40] Austys D., Dobrovolskij A., Jablonskienė V., Dobrovolskij V., Valevičienė N., Stukas R. (2019). Epicardial Adipose Tissue Accumulation and Essential Hypertension in Non-obese Adults. Medicina.

[41] van Woerden G., van Veldhuisen D.J., Rienstra M., Westenbrink B.D. (2020). Myocardial Adiposity in Heart Failure with Preserved Ejection Fraction: The Plot Thickens. Eur. J. Heart Fail..

[42] Wu C.-K., Lee J.-K., Hsu J.-C., Su M.-Y.M., Wu Y.-F., Lin T.-T., Lan C.-W., Hwang J.-J., Lin L.-Y. (2020). Myocardial Adipose Deposition and the Development of Heart Failure with Preserved Ejection Fraction. Eur. J. Heart Fail..

[43] Obokata M., Reddy Y.N.V., Pislaru S.V., Melenovsky V., Borlaug B.A. (2017). Evidence Supporting the Existence of a Distinct Obese Phenotype of Heart Failure With Preserved Ejection Fraction. Circulation.

[44] Rabkin S.W., Campbell H. (2015). Comparison of Reducing Epicardial Fat by Exercise, Diet or Bariatric Surgery Weight Loss Strategies: A Systematic Review and Meta-Analysis. Obes. Rev..

[45] Fenk S., Fischer M., Strack C., Schmitz G., Loew T., Lahmann C., Baessler A. (2015). Successful Weight Reduction Improves Left Ventricular Diastolic Function and Physical Performance in Severe Obesity. Int. Heart J..

[46] Jin X., Hung C., Tay W.T., Soon D., Sim D., Sung K., Loh S.Y., Lee S., Jaufeerally F., Ling L.H. (2022). Epicardial Adipose Tissue Related to Left Atrial and Ventricular Function in Heart Failure with Preserved versus Reduced and Mildly Reduced Ejection Fraction. Eur. J. Heart Fail..

[47] Pieske B., Tschöpe C., De Boer R.A., Fraser A.G., Anker S.D., Donal E., Edelmann F., Fu M., Guazzi M., Lam C.S.P. (2019). How to Diagnose Heart Failure with Preserved Ejection Fraction: The HFA-PEFF Diagnostic Algorithm: A Consensus Recommendation from the Heart Failure Association (HFA) of the European Society of Cardiology (ESC). Eur. Heart J..

[48] Kanagala P., Arnold J.R., Singh A., Chan D.C.S., Cheng A.S.H., Khan J.N., Gulsin G.S., Yang J., Zhao L., Gupta P. (2020). Characterizing Heart Failure with Preserved and Reduced Ejection Fraction: An Imaging and Plasma Biomarker Approach. PLoS ONE.

[49] Linde C., Ekström M., Eriksson M.J., Maret E., Wallén H., Lyngå P., Wedén U., Cabrera C., Löfström U., Stenudd J. (2022). Baseline Characteristics of 547 New Onset Heart Failure Patients in the PREFERS Heart Failure Study. ESC Heart Fail..

[50] Bosch L., Lam C.S.P., Gong L., Chan S.P., Sim D., Yeo D., Jaufeerally F., Leong K.T.G., Ong H.Y., Ng T.P. (2017). Right Ventricular Dysfunction in Left-Sided Heart Failure with Preserved versus Reduced Ejection Fraction. Eur. J. Heart Fail..

[51] Aschauer S., Kammerlander A.A., Zotter-Tufaro C., Ristl R., Pfaffenberger S., Bachmann A., Duca F., Marzluf B.A., Bonderman D., Mascherbauer J. (2016). The Right Heart in Heart Failure with Preserved Ejection Fraction: Insights from Cardiac Magnetic Resonance Imaging and Invasive Haemodynamics. Eur. J. Heart Fail..

[52] Mohammed S.F., Hussain I., AbouEzzeddine O.F., Takahama H., Kwon S.H., Forfia P., Roger V.L., Redfield M.M. (2014). Right Ventricular Function in Heart Failure with Preserved Ejection Fraction: A Community-Based Study. Circulation.

[53] Haase V.H. (2013). Regulation of Erythropoiesis by Hypoxia-Inducible Factors. Blood Rev..

[54] Shi K.-L., Qi L., Mao D.-B., Chen Y., Qian J.-Y., Sun Y.-B., Guo X.-G. (2015). Impact of Age on Epicardial and Pericoronary Adipose Tissue Volume. Eur. Rev. Med. Pharmacol. Sci..

[55] Silaghi A., Piercecchi-Marti M.D., Grino M., Leonetti G., Alessi M.C., Clement K., Dadoun F., Dutour A. (2008). Epicardial Adipose Tissue Extent: Relationship with Age, Body Fat Distribution, and Coronaropathy. Obesity.

[56] Rabkin S. (2022). Evaluating the Adverse Outcome of Subtypes of Heart Failure with Preserved Ejection Fraction Defined by Machine Learning: A Systematic Review Focused on Defining High Risk Phenogroups. EXCLI J..

